# Characterization of Internal Medicine Chief Resident Administrative, Educational, and Clinical Experiences

**DOI:** 10.1001/jamanetworkopen.2022.3882

**Published:** 2022-03-23

**Authors:** Megha Garg, Tejaswi Kompala, Michael Hurley, Lenny López

**Affiliations:** 1 Department of Medicine, University of California, San Francisco; 2 San Francisco Veterans Affairs Medical Center, San Francisco, California; 3 Teladoc Health, Mountain View, California

## Abstract

**Importance:**

The internal medicine (IM) chief residency is a position of leadership and honor common in IM programs, but the goals, responsibilities, and experiences of those who undertake it can be highly variable. Studies assessing the experience and impressions of the chief resident (CR) position from the viewpoint of the IM CRs are lacking.

**Objective:**

To describe the structure, responsibilities, and perceptions of the IM CR role across IM residency programs.

**Design, Setting, and Participants:**

A cross-sectional, simple descriptive electronic survey for current CRs was administered between April and June 2018 across US IM residency programs accredited by US Accreditation Council for Graduate Medical Education. A 2-step nonrandom sampling approach was used: first, snowball sampling was performed using the authors’ professional networks, and second, the survey was sent to the Association of Program Directors in Internal Medicine (APDIM) CR listserv. Data analysis was performed from June 2020 to August 2020.

**Exposures:**

Participation as a CR for an IM residency program in the 2017 to 2018 academic year.

**Main Outcomes and Measures:**

Descriptive CR personal and program characteristics and CR perceptions of administrative, clinical, and leadership experiences.

**Results:**

Among 169 unique responses, 77 participants (46%) were female and 89 (53%) were White. The response rate was 57% (70 of 122 surveys) in the snowball sample and 12% (99 of 842 surveys) in the APDIM listserv. The 2 sampled groups were combined for analysis. Most respondents (125 CRs [74%]) were from academic or university-based programs. Common across CR responses was responsibility for administrative tasks, clinical work, and educational efforts. Most CRs (111 of 157 respondents [71%]) reported being the primary schedulers for the residency program. Clinical work differed widely across respondents. Only 70 of 156 respondents (45%) reported having an academic title associated with the CR role. CRs reported inconsistent evaluation throughout the year, with high percentages reporting never receiving feedback on teaching (34 respondents [23%]), clinical abilities (67 respondents [45%]), or leadership abilities (60 respondents [40%]). Most CRs (107 respondents [69%]) agreed that they find work as a CR fulfilling and 117 (74%) agreed they would do chief residency again.

**Conclusions and Relevance:**

Despite its ubiquity in training programs across the US, the IM CR experience is very different across programs. Recommendations are provided to consider for improvement of the CR experience, including structured feedback opportunities, maximizing educational and clinical experiences, and standardizing policies.

## Key Points

**Question:**

What is the experience of the internal medicine chief residency from the perspective of the chief resident?

**Findings:**

In this national cross-sectional survey of 169 internal medicine chief residents, 71% were the primary schedulers for their programs, clinical work differed widely across respondents, and substantial numbers of chief residents reported that they never received feedback on teaching, clinical work, or leadership. Most (74%) reported that they would do chief residency again.

**Meaning:**

These findings suggest that the internal medicine chief residency experience is highly variable and could benefit from structured feedback opportunities and standardized policies.

## Teaser

This cross-sectional survey study assesses the perceptions of internal medicine chief residents regarding their administrative, educational, and clinical experiences in the chief residency role.

## Introduction

The internal medicine (IM) chief residency is regarded as a position of leadership and honor in IM programs. It is a multifaceted role that includes education, administration, and clinical duties. Although the chief resident (CR) position is ubiquitous across IM programs, the goals, responsibilities, and experiences of those who undertake it can be highly variable because of institutional tradition, resource allocation, and program needs and expectations. Although there exist training programs for CRs across specialties^1^ and specifically for IM,^2^ the Accreditation Council for Graduate Medical Education outlines minimal chief residency requirements in the Common Program Requirements. There are only 2 stipulations for CRs who have not yet completed their residency (postgraduate year [PGY] 3 chiefs); first, they are allowed a small increase of duty hour limits, and second, PGY3 chiefs must not contribute to the clinical promotion recommendations for resident peers in the Clinical Competency Committee.^3^

Specialties other than IM have explored the administrative, leadership, education, and clinical aspects of the CR role.^4,5,6^ One survey^4^ of emergency medicine CRs and program directors found that the program directors reported more CR job training and more CR influence on the residency program than did the CRs. Studies of the anesthesiology and physiatry chief residencies found variation across institutions in selection process.^5,6^ There are few published studies focused on the demographics, experience, and responsibilities of IM CRs. One study^7^ assessed the IM chief residency from the program director perspective, reporting that 40% of CRs were women, programs had a mean (SD) of 2.5 (1.4) CRs, and the mean annual salary was $60 000. There was substantial variability in the amount and type of clinical service, academic rank, and scholarship expectations across programs. Another, earlier study^8^ found that the burden of administrative work was increasing for CRs and that this was correlated with decreased satisfaction with the role. Some studies have looked at the leadership experience of CRs, including the unique challenges of coleadership in chief residency, as multiple people can be tasked with overlapping responsibilities.^9,10,11^ The IM CR role has been described as a “middle manager” who navigates the requirements of their institutional leadership as well as relationships with the residents.^11^

A common theme across studies is that those who have done a chief residency are more likely to become teaching faculty or leaders in their profession during their careers.^8,9,12^ Given the CR’s essential role in the functioning of a residency program and the future potential for academic leadership, it is important to better understand the role of IM CRs and the skills they obtain through a chief residency year. To our knowledge, there is no current study assessing the experience and impressions of the CR position from the viewpoint of the IM CRs. We sought to better elucidate the structure and responsibilities of the IM CR across US IM residency programs through a cross-sectional national survey, including their reflections and opinions about the experience. After reviewing the responses, we propose recommendations for best practices and opportunities for improvement of the IM CR role.

## Methods

We created an anonymous, electronic survey for current CRs in the 2017 to 2018 academic year administered between April and June 2018, near the completion of the chief residency academic year. Participants were not compensated for completion of the survey, and this study was exempt after review by the University of California, San Francisco institutional review board because it was a survey study in which survey participants could not be identified. The survey followed American Association for Public Opinion Research (AAPOR) reporting guidelines for survey studies.^13^

Two coauthors (T.K., then a current CR, and M.G., a former CR, from different institutions) created the survey using themes drawn from the literature review, a prior published survey,^7^ and their individual experiences. The survey was piloted for clarity and understandability (cognitive testing) with 10 former CRs known to the authors across various institutions and was revised according to their feedback. These former CRs were not participants in the current study.

The survey comprised 62 questions over 11 sections, including demographics, program structure, policies and compensation, responsibilities, work policies, performance evaluation and perceptions. Question types included multiple choice, 7-point Likert scale, and open-ended response. Respondents chose their race or ethnicity from the following categories: White; African American or Black; Asian; American Indian or Alaska Native; Native Hawaiian or Pacific Islander; Hispanic or Latino(a); or Prefer not to answer. They could select more than one answer. Race and ethnicity were collected in this study as a standard demographic variable. The survey was administered online using a Qualtrics platform. See the eAppendix in the Supplement for the complete survey.

Currently, there is no centralized national database or listserv that contains contact information for all IM CRs nationally. Random samples cannot be selected when the size of the population is unknown, individuals cannot be easily identified, access to the potential respondents is restricted, or contact information is unattainable. Therefore, we used a 2-step nonrandom sampling approach from the same CR population to maximize sample size. First, we used a snowball sampling method to generate a list of CR email contact information starting with the authors’ professional networks. This process resulted in 122 CRs across 30 IM programs. Second, we sent the finalized survey to the Association of Program Directors in Internal Medicine (APDIM) CR listserv for additional CR recruitment. At the time in 2018, the listserv had 842 members, but this number does not represent the true total number of IM CRs at that time because it also included some who had yet to start their chief year and individuals who may have opted out of email notification. Three waves of email invitations over 8 weeks were sent to maximize the response rate.

### Statistical Analysis

Our hypothesis is that our outcomes (survey question responses) vary by individual demographic and program characteristics. Each CR is expected to have unique perceptions of their CR experiences. As such, we would expect that there is individual level variation for outcomes. We tested for differences from our 2 nonrandom samples to assess for similarity in individual demographic and program characteristics.^14^ Our study is a simple descriptive survey using a nonrandom sampling approach. As noted earlier, we were unable to accurately define the complete population of CRs. As a result, we were unable to define the probabilities of sample selection from the population, which prevents inferential statistics. Thus, our analyses were restricted to descriptive statistics and we did not provide statistical inference.^15,16^ We assessed the associations among categorical variables from the survey results using the χ^2^ test or Fisher exact test where appropriate. Significance was set at *P* < .05. Our findings are hypothesis generating and not causal. Descriptive statistics of frequencies and percentages were performed with Excel version 16.0 (Microsoft) and Stata MP statistical software version 13.1 (StataCorp). Data were analyzed from June 2020 to August 2020.

## Results

### Characteristics of Snowball vs Listserv Samples

We received 169 unique responses to our survey. Of the snowball sample, our response rate was 57% (70 of 122 surveys). From the APDIM listserv, we received 99 of 842 surveys (12%). The race and ethnicity (50 White respondents [50%] in the APDIM sample vs 41 White respondents [59%] in the snowball sample) and gender identity percentages (45 female respondents [46%] in the APDIM sample vs 32 female respondents [46%] in the snowball sample) within responding groups were not meaningfully different. The 2 surveyed groups differed in 2 demographic characteristics: (1) the number of CRs from an academic or university-based program (64 respondents [64%] in the APDIM group vs 61 respondents [88%] in the snowball sample, *P* < .001, Fisher exact test), and (2) the number of CRs who graduated from US medical schools (72 respondents [72%] in the APDIM sample vs 60 respondents [87%] in the snowball sample, χ^2^_1_ = 5.341; *P* = .02). Given the relative similarity between the 2 sample groups and the intention to describe the largest number of IM CRs possible, the 2 sampled groups were combined. We report the results of 169 responses below; blank answers were excluded from the analysis when applicable.

### Demographics

Survey respondents represented a range of IM program sizes with nearly equal distribution across small, medium, and large programs (Table 1). Approximately one-half of the respondents (77 respondents [46%]) identified as female (the reported CR gender identity by APDIM in 2017-2018 was 42.8% female). The majority (89 respondents [53%]) identified as White. Most respondents (125 CRs [74%]) were from academic or university based programs. More than one-half of respondents reported future plans for fellowship, with 41% (69 respondents) already accepted to a fellowship and an additional 20% (33 respondents) planning to apply. Eighty percent were PGY4 CRs (135 respondents). Of those who reported a defined CR role, 23% (39 respondents) were inpatient and 14% (24 respondents) were quality improvement/patient safety focused. Most (86 respondents [51%]) reported a mixed CR role. The most common program size represented was 101 to 150 residents (34%).

**Table 1.** Survey Respondent Characteristics

Characteristic	Respondents, No. (%) (N = 169)^a^
Type of program	
Academic or university based	125 (74)
Community based	40 (24)
Military or uniformed health service based	4 (2)
Gender identity	
Female	77 (46)
Male	92 (54)
Other	0
Race and ethnicity	
African American or Black	8 (5)
African American or Black and Hispanic or Latino(a)	1 (1)
American Indian or Alaska Native	2 (2)
Asian	44 (26)
White	89 (53)
Hispanic/Latino(a)	19 (11)
Fellowship plans	
Already accepted to fellowship	69 (41)
Planning to apply	33 (20)
Returning in between fellowship years to do my chief year	2 (1)
None	58 (34)
Undecided	7 (4)
PGY	
PGY 3	25 (15)
PGY 4	135 (80)
PGY 5	8 (5)
PGY 6 and beyond	1 (1)
Chief resident role	
Ambulatory or primary care	12 (7)
Inpatient	39 (23)
Mixed	86 (51)
Quality improvement or safety	24 (14)
No answer	8 (5)
Size of program, No. of residents	
1-50	28 (17)
51-100	44 (26)
101-150	58 (34)
>150	38 (22)
No answer	1 (1)

Abbreviation: PGY, postgraduate year.

^a^ Totals may not equal 100% because of blank responses.

### Responsibilities

Common across most CR responses was responsibility for administrative tasks, clinical work, and educational efforts.

Regarding administrative tasks, 71% of CRs (111 of 157 respondents) reported that they are the primary people responsible for resident schedules. Almost all CRs (150 of 157 CRs [96%]) were responsible for emergency scheduling at their institution. In free response, CRs described other administrative tasks they complete, including room reservations, organizing or ordering food, technical support, and maintaining online information.

Clinical work was variable among CRs. Only 12% of CRs (19 of 155 respondents) reported no inpatient attending work, and conversely 14% (22 of 155 respondents) worked greater than 11 weeks inpatient. The plurality (53 of 155 respondents [34%]) worked between 7 and 10 weeks inpatient. More than half (96 of 146 respondents [55%]) indicated that they precept in an outpatient clinic, whereas only 27% (39 of 143 respondents) maintained their own outpatient primary care panel for the year. Of those who precepted residents in outpatient clinic, 75% (70 of 95 respondents) did so 0 to 1 time per week. Most clinical work was with students or residents present, as 61% (91 of 149 respondents) reported never doing clinical work independently without learners.

Educationally, CRs affirmed traditional tasks associated with the role including running morning reports, morbidity and mortality conferences, medical student education, and board preparation. Of those who described morning reports further, approximately one-half (73 of 152 respondents [48%]) reported always preparing them ahead of time, whereas 16% (24 of 152 respondents) were spontaneous or unscripted, and the remainder (55 of 152 respondents [36%]) were a mix of the 2. CRs additionally reported involvement in a number of leadership activities including hospital committees, quality improvement, resident remediation, wellness, recruitment, and programmatic development for residents.

These responsibilities reflect some of the reasons they indicated choosing to do a CR year, including preparation for an academic career, honor and prestige, leadership opportunities, and competitiveness for fellowship.

### Policies and Compensation

Table 2 outlines reported compensation including salary, discretionary funds, vacation and parental leave policies, academic title, and moonlighting policies. Findings include those reporting that there is no specific vacation policy (30 of 158 respondents [19%]), uncertainty about parental leave policy (77 of 160 respondents [48%]), and only 45% (70 of 156 respondents) having an academic title. Those with an academic title usually indicated “Clinical Instructor” or “Assistant Professor” as the title. CRs also reported work hours, with most describing their workload to be 41 to 50 hours per week (58 of 161 respondents [36%]) or 51 to 60 hours per week (47 of 162 respondents [29%]).

**Table 2.** Work Policies and Compensation

Policies and Compensation	Respondents, No. (%)^a^
How many hours per week do you work on average?	
<30 h	1 (1)
30-40 h	28 (18)
41-50 h	58 (36)
51-60 h	47 (29)
> 60 h	26 (16)
No response	9
How much vacation do you get?	
1-2 wk	4 (3)
3-4 wk	121 (76)
5-6 wk	5 (3)
No specific vacation policy	30 (19)
No response	9
Parental leave policy?	
I'm not sure	77 (48)
No	35 (22)
Yes	48 (30)
No response	9
What is your annual salary?	
<$50 000	4 (3)
$50 000 to <$75 000	75 (48)
$75 000 to <$100 000	65 (41)
$100 000 to <$125 000	8 (5)
$125 000 to <$150 000	2 (1)
Greater than $150 000	3 (2)
No response	12
Are you allowed to moonlight?	
I'm not sure	2 (1)
No	16 (10)
Sometimes	3 (2)
Yes	136 (87)
No response	12
Do you have discretionary funds to spend on chief resident projects or expenses?	
I'm not sure	24 (15)
No	62 (40)
Yes	70 (45)
No response	13
Do you have an academic title?	
I'm not sure	30 (19)
No	56 (36)
Yes	70 (45)
No response	13

^a^ Blank responses were excluded from analysis.

### Performance Evaluation

CRs reported inconsistent feedback and evaluation throughout the year (Table 3). High percentages of CRs reported never receiving feedback on teaching (34 respondents [23%]), leadership abilities (60 respondents [40%]), clinical abilities (67 respondents [45%]), administrative abilities (75 respondents [50%]), interpersonal skills (74 respondents [49%]), or professionalism (71 respondents [47%]), When feedback was received, the plurality of responses indicated that it was once or twice yearly in any of these given areas (ranging from 31% to 45%). Less than 5% of respondents reported receiving weekly feedback in any of these areas.

**Table 3.** Frequency of Performance Evaluation During Chief Residency

Evaluation criteria	Respondents, No. (%)^a^				
	Never	Once or twice yearly	Quarterly or monthly	Weekly	Total No.
Teaching abilities	34 (23)	68 (45)	42 (28)	6 (4)	150
Leadership abilities	60 (40)	60 (40)	27 (18)	3 (2)	150
Clinical skills	67 (45)	53 (35)	28 (19)	2 (1)	150
Administrative skills	75 (50)	55 (37)	18 (12)	1 (1)	149
Interpersonal skills	74 (49)	47 (31)	25 (17)	2 (1)	148
Professionalism	71 (47)	50 (33)	25 (17)	1 (1)	147

^a^ Blank responses were excluded from analysis.

### Perceptions

CRs generally responded positively to survey questions related to their perceptions of the experience. Most CRs (107 respondents [69%]) strongly agreed or agreed that they find work as a CR fulfilling, 117 (74%) said they would do chief residency again, 121 (77%) “feel supported by [their] program leadership,” and 120 (75%) “have a say in changes for [the] residency program.” Only one-half (79 respondents [50%]) responded that “chief residency is what [they] expected” (Table 4). When asked how likely they would be to recommend the CR job to a friend on a scale of 1 to 10, the majority (75%, 117 of 156) said 7 through 10, 16% (25) responded 5 through 6, and a minority (14 respondents [9%]) responded 1 through 4.

**Table 4.** Perceptions of Chief Resident Experience

Perception of experience	Respondents, No. (%)^a^				
	Strongly agree or agree	Somewhat agree	Neutral	Somewhat disagree	Strongly disagree or disagree
I find my work as a chief resident fulfilling	109 (69)	26 (16)	17 (11)	4 (3)	3 (2)
If I could go back in time, I would make the choice to do chief residency again	117 (74)	18 (11)	12 (8)	5 (3)	7 (4)
I feel supported by my program leadership (PDs, APDs, Site Directors)	121 (77)	17 (11)	7 (4)	8 (5)	5 (3)
I have a say in changes for my residency program	120 (75)	22 (14)	7 (4)	9 (6)	1 (1)
Chief residency is what I expected	79 (50)	45 (28)	11 (7)	14 (9)	9 (6)

Abbreviations: APD, associate program director; PD, program director.

^a^ Blank responses were excluded from analysis.

## Discussion

Despite its ubiquity in training programs across the US, the IM CR experience is highly variable according to our survey of CRs across the country. Nevertheless, teaching, administrative work, and clinical service remain core components of the role. The actual work described within these components is not standardized across institutions, but one consistent finding was the commonality of CRs being involved in scheduling (71% primary scheduling, 96% emergency scheduling).

The majority of CRs in our survey were in the PGY4 of training and had plans to pursue a fellowship after the chief residency year. Perceptions of the experience were mainly positive, indicated by the number of CRs who would do the job again (74%) and felt supported by residency leadership (77%). This was similar to previous findings in a study among emergency medicine CRs.^4^ Three-quarters (75%) would very likely recommend the CR job to a friend. However, we identified several gaps and opportunities for improvement in the CR experience. We offer commentary on several points of interest that may be amenable to future policy and practice changes to enhance the IM chief residency experience.

First, a notably high number of CRs reported that they never received feedback on their performance across multiple domains. Of the identified core components of the role, 23% percent never received feedback on teaching, 45% responded “never” on clinical skills, and 50% said “never” on administrative skills. Less than 30% reported receiving quarterly or more frequent feedback in any domain, including the aforementioned domains as well as leadership, interpersonal skills, and professionalism. For a year of training that is intended to support the development of future academic physicians, this is a serious missed opportunity. Of note, program directors in a previous study^7^ reported evaluating their chiefs quarterly or monthly in teaching in more than 50% of programs, which demonstrates a difference in perception among CRs and program directors. This is not unique to IM. CRs in a qualitative study across specialties identified a desire for more formal feedback.^10^ These findings suggest a need for IM programs to ensure formal, structured feedback opportunities for CRs.

Additionally, both formal and free-response answers throughout our survey suggested that the most meaningful aspects of the job are focused on education, clinical, and leadership skills. Although managerial tasks are arguably a necessary component of administrative leadership, it is worth assessing which specific tasks are most valuable for future professional roles vs what may be outsourced to other administrative staff. Future studies may also explore the structure of programs that do not use CRs as primary schedulers for the residency.

Another finding from our survey is the lack of clarity of procedures and policies during the CR year. This included no formal understanding of vacation policies, parental leave policies, or discretionary fund availability. The CR year could be viewed as a hybrid training and formal first career position. Outlining basic policies would be a standard expectation of any job. These are policies that could be easily made available and reviewed at the outset of a CR year to allow for clear expectations and benefits of the role and to allow a potential CR to better evaluate the benefits of the CR year compared with other job opportunities.

Regarding the formal educational rank of the CR role, fewer than one-half (45%) of CRs reported having an academic title in affiliation with their role, similar to what was previously reported in the literature.^7^ Given that those who complete a CR are more likely to become academic faculty in the future, an academic title could provide added incentive for those interested in the position.^8^ Although it may use staff and time resources of an institution, providing an academic title does not cost the institution any additional salary for a CR, and could benefit future CR careers.

Finally, although CRs report generally positive experiences with the role, there are no current guidelines for clinical hours, learning opportunities, and equity concerns for chief residency. The role of the IM CR has not substantially changed since it was described in 1982,^8^ yet 50% of current survey respondents stated that “chief residency is not what they expected.” This interesting paradox may reflect a lack of standardized protocols for the role, and should be explored in future research studies. Given the ubiquity of the CR role across IM programs, it is worth considering formal guidance and mandated standards for the role from the Accreditation Council for Graduate Medical Education.

### Limitations

There are several limitations to this study. Currently, there is no centralized up-to-date database of CRs across the country. As a result, we used a 2-pronged sampling approach consisting of a convenience snowball sample and a listserv-based sample. Nonetheless, a review of the published literature suggests that this survey captured the largest number of CR perspectives to date across any specialty. Because the survey was anonymous, we do not know how many distinct programs are represented in the responses, as multiple CRs from the same program may have answered the survey. However, we believe that capturing the responses from different CRs at the same program better achieves the goal of capturing the individual CR view of the position. Additionally, our results are weighted toward academic residency programs (74%). Future studies should oversample from community-based IM residency programs.

## Conclusions

This study demonstrates the variability in the IM chief residency and identifies the perceived strengths and weaknesses of the experience. We provide recommendations to consider for improvement of the CR experience, including structured feedback opportunities, maximizing educational and clinical experiences, clear policies, provision of an academic title, and consideration of making the IM CR an Accreditation Council for Graduate Medical Education accredited year. Our survey provides a descriptive base for future studies of the chief residency experience and policy change.

## Notes

Supplement. **eAppendix.** Survey
