# Supplementary material for: Characterization of Internal Medicine Chief Resident Administrative, Educational, and Clinical Experiences
Source: JAMA Netw Open. 2022 Mar 23;5(3):e223882. doi: 10.1001/jamanetworkopen.2022.3882 (PMC8943621; doi:10.1001/jamanetworkopen.2022.3882)
Supplement: Supplement. — eAppendix. Survey [file jamanetwopen-e223882-s001.pdf]

Supplemental Online Content

Garg M, Kompala T, Hurley M, López L. Characterization of internal medicine chief resident administrative, educational, and clinical experiences. *JAMA Netw Open*. 2022;5(3):e223882.
doi:10.1001/jamanetworkopen.2022.3882

eAppendix. Survey

This supplemental material has been provided by the authors to give readers additional information about their work.

© 2022 Garg M et al. *JAMA Network Open.*

Chief Resident Survey - Purposeful Sample

Start of Block: Demographics

Thank you for taking this survey to inform the academic community about the roles of chief residents around the country! We hope this information will be useful to the medical education community. We expect the survey to take 8-10 minutes to complete. Please email Megha Garg (megha.garg@ucsf.edu) with any questions.

Please create a code using the first letters of your birth month followed by the last four digits of your phone number (ie birth month September, phone number 554-6677 would be SE6677). This will only be used to identify duplicate surveys and will NOT otherwise identify any survey participant.

____

At what type of program are you a chief resident (CR)?

◯ Academic/university based program (1)

◯ Community based program (2)

◯ Military/uniformed health service based program (3)

◯ Other: Please describe (4) ____

What post graduate year are you?

◯ PGY 3 (1)

◯ PGY 4 (2)

◯ PGY 5 (3)

◯ PGY 6 and beyond (4)

© 2022 Garg M et al. *JAMA Network Open.*

What is your current gender identity?

- ○ Male (1)
- ○ Female (2)
- ○ Transgender male/trans man/female-to-male (FTM) (3)
- ○ Transgender female/trans woman/male to female (MTF) (4)
- ○ Genderqueer, neither exclusively male nor female (5)
- ○ Additional gender category, please specify: (6) ____
- ○ Choose not to disclose (7)

What is your age?

- ○ 25 or under (1)
- ○ 26-30 (2)
- ○ 31-35 (3)
- ○ >35 (4)
- ○ Choose not to answer (5)

Where did you graduate from medical school?

- ○ United States/US Territory (1)
- ○ Outside of the United States/International (2)

© 2022 Garg M et al. *JAMA Network Open.*

What is your race/ethnicity?

- ☐ Caucasian (1)
- ☐ African American / Black (2)
- ☐ Hispanic / Latino(a) (3)
- ☐ American Indian / Alaska Native (4)
- ☐ Native Hawaiian / Pacific Islander (5)
- ☐ Asian (6)
- ☐ Prefer not to answer (7)

Do you identify with any of the following sexual orientations?

- ○ Straight or heterosexual (1)
- ○ Lesbian, gay, or homosexual (2)
- ○ Bisexual (3)
- ○ Something else (4)
- ○ Don't know (5)
- ○ Choose not to answer (6)

© 2022 Garg M et al. *JAMA Network Open.*

Are you applying to fellowship?

- ○ Yes (1)
- ○ No (2)
- ○ Undecided (3)
- ○ Already accepted to fellowship (4)
- ○ I am returning in between fellowship to do my chief year (5)
- ○ Already completed fellowship (6)

Display This Question:

If Are you applying to fellowship? = Yes

Or Are you applying to fellowship? = Already accepted to fellowship

Or Are you applying to fellowship? = I am returning in between fellowship to do my chief year

What fellowship are you doing or planning to do?

____

End of Block: Demographics

Start of Block: Program Structure

What category of chief residents exist at your institution? Select all that apply.

- ▢ Inpatient CR (1)
- ▢ Ambulatory / Primary Care (2)
- ▢ QI / Safety (3)
- ▢ Mixed (4)
- ▢ Other (5)
- ▢ N/A (6)

© 2022 Garg M et al. *JAMA Network Open.*

What is your chief resident role?

- ◯ Inpatient (1)
- ◯ Ambulatory / Primary Care (2)
- ◯ QI / Safety (3)
- ◯ Mixed (4)
- ◯ Other (5)
- ◯ N/A (6)

Display This Question:

If What is your chief resident role? = Other

Or What is your chief resident role? = Mixed

What is your chief resident role?

____

Display This Question:

If What is your chief resident role? = QI / Safety

Are you a VA Quality and Patient Safety Chief Resident?

- ◯ Yes (1)
- ◯ No (2)
- ◯ I don't know (3)

© 2022 Garg M et al. *JAMA Network Open.*

How were you selected to be a chief resident?

◯ I applied through a formal application process (1)

◯ I was selected by my residency program leadership without a formal application (2)

◯ Other process (please describe) (3) ____

Are you serving as a Chief Resident at the same institution where you did residency?

◯ Yes, same institution (1)

◯ No, I trained at a different institution (2)

How many chief residents are at your institution?

◯ 1 (1)

◯ 2-4 (2)

◯ 5-7 (3)

◯ >7 (4)

Display This Question:

If How many chief residents are at your institution? != 1

If your institution has >1 chief resident, do you have the same responsibilities as your co-chief resident(s)?

◯ Yes (1)

◯ No (2)

Display This Question:

If How many chief residents are at your institution? != 1

And If your institution has 1 chief resident, do you have the same responsibilities as your co-chief... = No

© 2022 Garg M et al. *JAMA Network Open.*

How do your responsibilities differ from your co-chiefs (please check all that apply):

- ☐ We work at different clinical sites (1)
- ☐ We have different administrative responsibilities (scheduling, backup call, etc) (2)
- ☐ We have a different focus of work (inpatient, outpatient, quality, etc) (3)
- ☐ Other (4) ____

How many number of total residents (PGY1-PGY3) are at your institution?

- ○ 1-50 (1)
- ○ 51-100 (2)
- ○ 101-150 (3)
- ○ >150 (4)
- ○ I'm not sure (5)

How many hospitals/clinical sites do your residents work at?

- ○ 1 (1)
- ○ 2 (2)
- ○ 3 (3)
- ○ 4 or more (4)

Display This Question:

If How many hospitals/clinical sites do your residents work at? != 1

© 2022 Garg M et al. *JAMA Network Open.*

If your program has more than one hospital or clinical site, do you personally rotate between sites in your chief resident role?

○ Yes (1)

○ No (2)

○ Sometimes (3)

End of Block: Program Structure

Start of Block: Opinions and Work-Life Balance

© 2022 Garg M et al. *JAMA Network Open.*

Please answer the following questions:

	Strongly Agree (1)	Agree (2)	Somewhat Agree (6)	Neutral (3)	Somewhat Disagree (7)	Disagree (4)	Strongly Disagree (5)
I find my work as a chief resident fulfilling (1)	○	○	○	○	○	○	○
If I could go back in time, I would make the choice to do chief residency again (2)	○	○	○	○	○	○	○
I feel supported by my program leadership (PD, APDs, Site Directors) (3)	○	○	○	○	○	○	○
I have a say in changes for my residency program (4)	○	○	○	○	○	○	○
Chief Residency is what I expected (5)	○	○	○	○	○	○	○

Please elaborate if you disagreed with any of the above statements on why you chose your answer.

____

© 2022 Garg M et al. *JAMA Network Open.*

How much vacation do you get?

- 1-2 weeks (1)
- 3-4 weeks (2)
- 5-6 weeks (3)
- No specific vacation policy (4)

How many hours do you work per week on average?

- <30 hours (1)
- 30-40 hours (2)
- 41-50 hours (3)
- 51-60 hours (4)
- > 60 hours (5)

Is there an official parental leave policy for chief residents?

- Yes (1)
- No (2)
- I'm not sure (3)

© 2022 Garg M et al. *JAMA Network Open.*

When you are not attending on the inpatient service, do you work weekends in your CR role? (e.g. holding Jeopardy or sick call pager, transfer pagers, teaching or administrative work, etc)

- ◯ Yes (1)
- ◯ No (2)
- ◯ Sometimes (3)

End of Block: Opinions and Work-Life Balance

Start of Block: Training

Do you have job training or workshops in preparation for chief year?

- ◯ Yes (1)
- ◯ No (2)

Display This Question:

If Do you have job training or workshops in preparation for chief year? = Yes

What type of job training or workshops did you have in preparation for chief year? (Check all that apply)

- ▢ Meeting/training with outgoing chief (1)
- ▢ Written documentation of roles and expectations (2)
- ▢ Internal faculty led training or workshop (4)
- ▢ Attendance at conference or external meetings (3)
- ▢ Other (5) ____

© 2022 Garg M et al. *JAMA Network Open.*

Did your program support your attendance at the Association of Program Directors in Internal Medicine (APDIM) Chief Resident meeting? Select all that apply.

- ☐ Financial support (1)
- ☐ Service coverage support (2)
- ☐ Neither (3)
- ☐ I'm not sure (4)

Did you attend APDIM?

- ○ Yes (1)
- ○ No (2)

How much overlap did you have with previous year chiefs?

- ○ Days (1)
- ○ Weeks (2)
- ○ Months (3)
- ○ None (4)

End of Block: Training

Start of Block: Administrative

© 2022 Garg M et al. *JAMA Network Open.*

Are CRs the primary people responsible for resident scheduling at your institution?

- ○ Yes (1)
- ○ No (2)
- ○ Sometimes (4)
- ○ I'm not sure (3)

Are CRs the primary people responsible for emergency scheduling (eg resident illness, overcapped census, "jeopardy") at your institution?

- ○ Yes (1)
- ○ No (2)
- ○ Sometimes (4)
- ○ I'm not sure (3)

What kind of administrative tasks are you responsible for? Check all that apply.

- ☐ Organizing / ordering food (1)
- ☐ Room reservations (2)
- ☐ Cleaning (3)
- ☐ Photocopying (4)
- ☐ Purchasing supplies (5)
- ☐ Technical support / computer problems (6)
- ☐ Webmaster / maintaining online information (7)
- ☐ Other, please describe: (8) ____

© 2022 Garg M et al. *JAMA Network Open.*

End of Block: Administrative

Start of Block: Financial

What is your salary?

- < 50 K (1)
- 50K to < 75K (2)
- 75K to (3)
- 100 to (4)
- 125K to < 150K (5)
- Greater than 150K (6)

Are you paid on the PGY scale?

- Yes (1)
- No (2)
- I'm not sure (3)

Display This Question:

If Are you paid on the PGY scale? = Yes

If yes, what PGY Year salary do you receive? (eg PGY 4, PGY 8, etc)

- PGY (1) ____

© 2022 Garg M et al. *JAMA Network Open.*

Are you allowed to moonlight?

- Yes (1)
- No (2)
- Sometimes (4)
- I'm not sure (3)

Display This Question:

If Are you allowed to moonlight? = Yes

Or Are you allowed to moonlight? = Sometimes

Do you have a limit to how much you are allowed to moonlight?

- Yes. How much? (1) ____
- No (2)
- I'm not sure (3)

Do you have discretionary funds to spend on chief resident projects/expenses?

- Yes (1)
- No (2)
- I'm not sure (3)

Display This Question:

If Do you have discretionary funds to spend on chief resident projects/expenses? = Yes

© 2022 Garg M et al. *JAMA Network Open.*

If yes, how much discretionary funding do you have?

- ○ $1000 or less (1)
- ○ $1000 - $5000 (2)
- ○ Greater than $5000 (3)
- ○ I'm not sure (4)

End of Block: Financial

Start of Block: Attending Responsibilities

Do you have an academic title?

- ○ Yes (1)
- ○ No (2)
- ○ I'm not sure (3)

Display This Question:

If Do you have an academic title? = Yes

What academic title do you have?

- ○ Clinical Instructor (1)
- ○ Assistant Professor (2)
- ○ Adjunct Professor/Faculty (4)
- ○ Volunteer Faculty (5)
- ○ Other (3) ____

© 2022 Garg M et al. *JAMA Network Open.*

How many weeks per year do you attend on the inpatient medicine service?

- ○ No inpatient attending (1)
- ○ 1-2 weeks (2)
- ○ 3-4 weeks (3)
- ○ 5-6 weeks (4)
- ○ 7-10 weeks (5)
- ○ 11 + weeks (6)

Which of the following clinical responsibilities do you have?

	Yes (1)	No (2)	Sometimes (4)	N/A (3)
Precept residents in outpatient clinic (6)	○	○	○	○
Maintain your own outpatient primary care panel (7)	○	○	○	○
Perform clinical duties without learners (clinic or inpatient service) (8)	○	○	○	○

Display This Question:

If Which of the following clinical responsibilities do you have? = Precept residents in outpatient clinic [Yes]

Or Which of the following clinical responsibilities do you have? = Precept residents in outpatient clinic [Sometimes]

© 2022 Garg M et al. *JAMA Network Open.*

How many sessions per week of outpatient clinic do you precept?

- less than once weekly (5)
- 1 (1)
- 2-3 (2)
- 4-5 (3)
- 6 or more (4)

End of Block: Attending Responsibilities

Start of Block: Leadership Responsibilities

Are you involved in any of the following activities? Select all that apply.

- Quality Improvement (1)
- Remediation for residents or medical students (2)
- Resident recruitment (3)
- Resident wellbeing support (4)
- Programmatic development for residency (5)
- Curriculum development (6)
- Hospital committees (8)
- Other (7) ____

End of Block: Leadership Responsibilities

Start of Block: Educational Responsibilities

© 2022 Garg M et al. *JAMA Network Open.*

What types of educational activities do you lead? Select all that apply.

- ▢ Morning Report (1)
- ▢ Morbidity and Mortality Conference (M&M) (2)
- ▢ Medical student education (3)
- ▢ Board Prep (4)
- ▢ Other (5) ____

On average, how many times per week do the CRs at your institution lead morning report?

- ○ 0 (1)
- ○ 1-2 (2)
- ○ 3-4 (3)
- ○ 5 (4)
- ○ >6 (5)

On average, what percentage of morning reports per week do you personally lead?

- ○ 0-25 % (1)
- ○ 26-50 % (2)
- ○ 51-75 % (3)
- ○ 76-100% (4)

© 2022 Garg M et al. *JAMA Network Open.*

Morning report cases are:

- ◯ Prepared ahead of time (1)
- ◯ Spontaneous "unscripted" (2)
- ◯ Mixed (3)

Display This Question:

If What types of educational activities do you lead? Select all that apply. = Board Prep

How much time do you devote to board review per week?

- ◯ under 3 hours (1)
- ◯ 3 hours or more (2)
- ◯ Changes based on need/site/timing (3)

End of Block: Educational Responsibilities

© 2022 Garg M et al. *JAMA Network Open.*

Start of Block: Feedback and Evaluation

Do you receive feedback and evaluation on your:

	How frequently?				Who evaluates you?					
	Never (1)	Once or twice yearly (2)	Quarterly/ Monthly (3)	Weekly (4)	Program Directors/ Assoc PDs (1)	Residents (2)	Dept Chair (3)	Other Faculty (4)	Med Students (5)	Admin Staff (6)
Teaching abilities (1)	▢	▢	▢	▢	▢	▢	▢	▢	▢	▢
Leadership abilities (2)	▢	▢	▢	▢	▢	▢	▢	▢	▢	▢
Clinical abilities (3)	▢	▢	▢	▢	▢	▢	▢	▢	▢	▢
Administrative abilities (4)	▢	▢	▢	▢	▢	▢	▢	▢	▢	▢
Interpersonal skills (5)	▢	▢	▢	▢	▢	▢	▢	▢	▢	▢
Professionalism (6)	▢	▢	▢	▢	▢	▢	▢	▢	▢	▢

End of Block: Feedback and Evaluation

Start of Block: Free Response

© 2022 Garg M et al. *JAMA Network Open.*

Why did you decide to do chief residency? Select all that apply.

- ▢ Competitiveness for fellowship (1)
- ▢ Leadership role (2)
- ▢ Honor / prestige (3)
- ▢ Develop skills as educator (4)
- ▢ Prepare for academic medicine career (5)
- ▢ Other (6) ____

What is your favorite aspect of chief residency?

____

What is your least favorite aspect of chief residency?

____

What would you change about the experience?

____

© 2022 Garg M et al. *JAMA Network Open.*

How likely would you be to recommend this job to a friend? 0 is very unlikely, 10 is very likely.

- ○ 0 (1)
- ○ 1 (2)
- ○ 2 (3)
- ○ 3 (4)
- ○ 4 (5)
- ○ 5 (6)
- ○ 6 (7)
- ○ 7 (8)
- ○ 8 (9)
- ○ 9 (10)
- ○ 10 (11)

Please share anything else you would like to tell us about your chief residency?

____

End of Block: Free Response

© 2022 Garg M et al. *JAMA Network Open.*
